# Monogenic Causes of Proteinuria in Children

**DOI:** 10.3389/fmed.2018.00055

**Published:** 2018-03-12

**Authors:** Onur Cil, Farzana Perwad

**Affiliations:** ^1^Department of Pediatrics, Division of Nephrology, University of California San Francisco, San Francisco, CA, United States

**Keywords:** nephrotic syndrome, children, proteinuria, steroid, immunosuppression, genetics

## Abstract

Glomerular disease is a common cause for proteinuria and chronic kidney disease leading to end-stage renal disease requiring dialysis or kidney transplantation in children. Nephrotic syndrome in children is diagnosed by the presence of a triad of proteinuria, hypoalbuminemia, and edema. Minimal change disease is the most common histopathological finding in children and adolescents with nephrotic syndrome. Focal segmental sclerosis is also found in children and is the most common pathological finding in patients with monogenic causes of nephrotic syndrome. Current classification system for nephrotic syndrome is based on response to steroid therapy as a majority of patients develop steroid sensitive nephrotic syndrome regardless of histopathological diagnosis or the presence of genetic mutations. Recent studies investigating the genetics of nephrotic syndrome have shed light on the pathophysiology and mechanisms of proteinuria in nephrotic syndrome. Gene mutations have been identified in several subcellular compartments of the glomerular podocyte and play a critical role in mitochondrial function, actin cytoskeleton dynamics, cell–matrix interactions, slit diaphragm, and podocyte integrity. A subset of genetic mutations are known to cause nephrotic syndrome that is responsive to immunosuppressive therapy but clinical data are limited with respect to renal prognosis and disease progression in a majority of patients. To date, more than 50 genes have been identified as causative factors in nephrotic syndrome in children and adults. As genetic testing becomes more prevalent and affordable, we expect rapid advances in our understanding of mechanisms of proteinuria and genetic diagnosis will help direct future therapy for individual patients.

## Introduction

Proteinuria is a common clinical presentation in children with chronic kidney disease (CKD). In the United States, glomerular diseases causing proteinuria account for 22% of CKD in children (1). Nephrotic syndrome is defined as a triad of proteinuria (urine protein to creatinine ratio >2 or urine dip-3 + protein), hypoalbuminemia (<2.5 g/dl), and edema (2). Nephrotic syndrome occurs in approximately 1–3 in 100,000 live births in the United States and 80% of children respond to steroid therapy (3). A significant number of children with nephrotic syndrome have known genetic mutations identified during their clinical course. Previously, steroid sensitive nephrotic syndrome (SSNS) was considered idiopathic in origin and unlikely to be caused by genetic mutations. However, recent evidence suggests that a subset of patients with genetic variants or mutations do respond to immunosuppressive therapy (4–7). With the advent of genetic testing, we expect to have a better understanding of the mechanisms for proteinuria in children and tailor therapy to individual patients with the goal of minimizing unnecessary exposure to immunosuppressive agents.

## Etiology and Classification

Several classification systems have been proposed for nephrotic syndrome in children based on etiology, histopathology, response to steroid therapy, and genetic diagnosis. Classification is problematic because nephrotic syndrome is largely a heterogenic disease with multiple complex pathogenic mechanisms. Second, large clinical studies to characterize and correlate histopathology, response to immunosuppression, and genetic diagnosis are lacking. Based on etiology, nephrotic syndrome is classified as primary (idiopathic) or secondary due to infection (malaria, hepatitis, and HIV), malignancy (Hodgkin’s and non-Hodgkin’s lymphoma, bronchogenic carcinoma, and colon carcinoma), or other causes. However, in children, secondary causes of nephrotic syndrome are very rare. Nephrotic syndrome diagnosed in the first 3 months of life is called congenital nephrotic syndrome and is caused by genetic mutations in vast majority of the patients (8). An international study of primary nephrotic syndrome in children was conducted between 1967 and 1974 in 24 clinics in North America, Europe, and Asia (9). Renal biopsies were performed before starting treatment in 521 children between 12 weeks and 16 years of age excluding infants who were diagnosed with congenital nephrotic syndrome. The distribution of patients among histopathological categories revealed that 76.6% had minimal change disease (MCD), 7.5% had membranoproliferative glomerulonephritis, and 6.9% had focal segmental glomerulosclerosis (FSGS). In addition, the above study findings suggested that a histopathological diagnosis is not required before initiating therapy in children as a majority of patients respond to steroid treatment. Therefore, nephrotic syndrome in children is currently classified as SSNS or steroid resistant (SRNS) based on response to therapy. Approximately 20% of children with nephrotic syndrome are steroid resistant but calcineurin inhibitors, rituximab and/or ACEI therapy can successfully induce complete or partial remission in a large number of patients with SRNS with and without known genetic mutations (10). The clinical features and genetics of SRNS was recently characterized in a large multicenter international study of 1,655 children (PodoNet registry cohort) (8). The age at diagnosis greatly influenced the likelihood of finding a genetic mutation. The proportion of patients with a genetic disease cause decreased with increasing manifestation age: from 66% in congenital nephrotic syndrome to 15–16% in schoolchildren and adolescents. Similar observations were made in another large international study of SRNS patients (11); in 1,783 unrelated families with SRNS, a single-gene mutation was identified in 29.5% of families with SRNS that manifested before 25 years of age. The fraction of families in whom a single-gene cause was identified inversely correlated with age of onset. To date, more than 50 genes have been identified and as genetic testing becomes more affordable and accessible in clinical settings, monogenic causes of nephrotic syndrome is expected to increase significantly in the future (12).

## Pathophysiology and Genetics of Nephrotic Syndrome

Glomerular ultrafiltrate lacks cellular and macromolecular components of plasma due to selective permeability of the glomerular filtration barrier that separates blood and urinary space (13). Glomerular filtration barrier is composed of three layers: fenestrated endothelium, glomerular basement membrane (GBM), and podocyte foot processes. GBM is a protein network formed by type IV collagen, laminin, nidogen, and negatively charged proteoglycans that are thought to repel serum proteins electrostatically (13, 14). Podocytes are epithelial cells outside the GBM and have an actin-based contractile apparatus which is critical to the formation of the complex architecture of foot processes (15). Foot processes are linked together by the slit diaphragm which is a vital component of the glomerular filtration barrier. Several proteins expressed in podocytes play important roles in signal transduction from slit diaphragm to podocytes (16).

Glomerular filtration barrier functions as a size and charge selective molecular sieve and under physiological conditions only allow water and some plasma solutes to pass from blood stream to the urinary space. The transport of albumin and other large plasma proteins to urinary space is largely prevented by GBM and slit diaphragm. The small amounts of albumin and plasma proteins that pass through the barrier are reabsorbed in the proximal tubule through the megalin–cubilin pathway. Disturbances in any component of the filtration barrier can cause severe proteinuria due to glomerular protein losses leading to nephrotic syndrome.

Mechanisms by which the filtration barrier is perturbed in nephrotic syndrome have been investigated for several decades. In patients with SSNS and SRNS who respond to immunosuppressive therapy, proteinuria is thought to be caused by an underlying immunological defect (17). In earlier studies, production of a circulating proteinuric factor due to T-cell dysfunction was put forth as the leading hypothesis (18). Increased albumin permeability was demonstrated in rat glomeruli incubated with serum from FSGS patients lending support to this hypothesis (19, 20). However, discovery of novel gene mutations identified in adults and children with nephrotic syndrome provide evidence for an inherent defect in the structural integrity and function of the glomerular filtration barrier as the pathogenic mechanism for proteinuria (21). Interestingly, a majority of patients with genetic mutations respond to immunosuppressive therapy suggesting that these medications regulate the structure and function of the filtration barrier rather than immune modulation. Whether these genetic factors play a role in the pathogenesis of nephrotic syndrome in patients without an identified genetic mutation is yet to be investigated.

**Figure 1:**
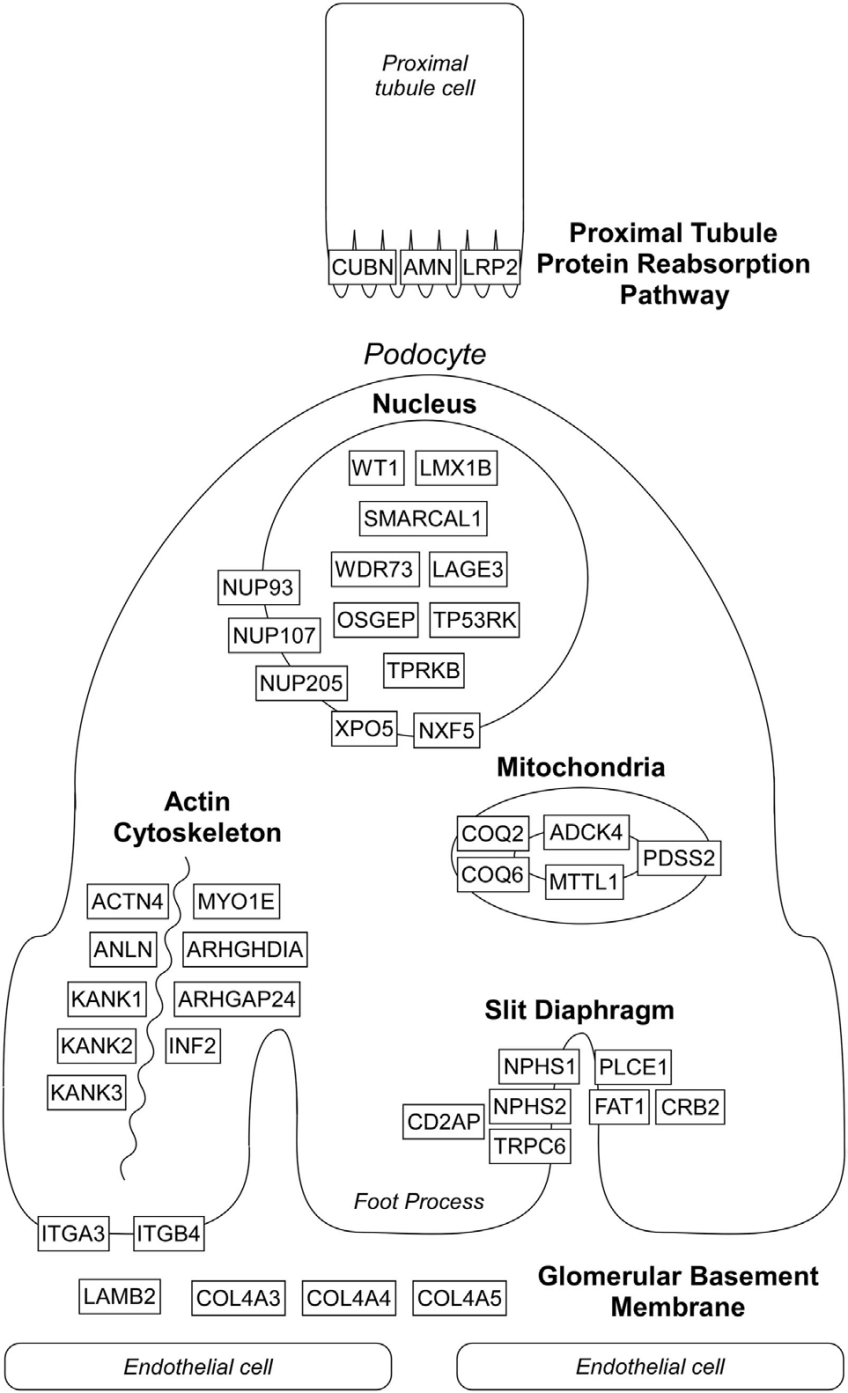
Localization of affected proteins in monogenic causes of proteinuria. Please see text and Table 1 for details. Diagram does not depict all genes listed in Table 1 known to cause proteinuria and nephrotic syndrome.

Over the past two decades, more than 50 monogenic causes of proteinuria have been identified which affect glomerular filtration barrier (slit diaphragm, podocyte actin cytoskeleton, adhesion, and GBM proteins), podocytes biology (mitochondria, nuclear transcription factors), and proximal tubule protein reabsorption pathways (Figure 1). Monogenic forms of proteinuria are summarized in Table 1. These genetic discoveries paved the way to a better understanding of the physiology and functions of the glomerular filtration barrier which can have therapeutic implications in the future.

**Table 1:** Monogenic forms of nephrotic syndrome and proteinuria.

Gene	Gene product	Inheritance	Associated syndrome/extrarenal findings	Reference
**Slit diaphragm related**				
*NPHS1*	Nephrin	AR		(21–27)
*NPHS2*	Podocin	AR		(28, 29)
*PLCE1*	Phospholipase Cε1	AR		(30, 31)
*CD2AP*	CD2-associated protein	AD/AR		(32)
*TRPC6*	Transient receptor potential cation channel type 6	AD		(33, 34)
*CRB2*	Crumbs homolog 2	AR		(35–37)
*FAT1*	FAT atypical cadherin 1	AR	Neurological involvement	(38–40)

**Actin cytoskeleton-related proteins**				
*ACTN4*	α-Actinin 4	AD		(41, 42)
*INF2*	Inverted formin 2	AD	Charcot–Marie–Tooth disease	(43, 44)
*MYO1E*	Non-muscle myosin 1E	AR		(45, 46)
*ANLN*	Anillin	AD		(47)
*ARHGAP24*	Rho GTPase-activating protein 24	AD		(48)
*ARHGDIA*	RhoGDP dissociation inhibitor α	AR	Seizures, intellectual disability	(49)
*KANK1*	Kidney ankyrin repeat-containing protein 1	AR		(6)
*KANK2*	Kidney ankyrin repeat-containing protein 2	AR		(6)
*KANK4*	Kidney ankyrin repeat-containing protein 4	AR		(6)

**Mitochondrial**				
*ADCK4*	aarF domain containing kinase 4	AR		(50–52)
*COQ2*	Coenzyme Q2 4-hydroxybenzoate polyprenyl transferase	AR	Seizures	(53)
*COQ6*	Coenzyme Q6 monooxygenase	AR	Sensorineural deafness	(54)
*MTTL1*	tRNA-LEU	Unknown	Mental retardation, diabetes mellitus, MELAS syndrome	(55, 56)
*PDSS2*	Prenyl diphosphate synthase subunit 2	AR	Encephalomyopathy, Leigh syndrome	(57)

**Glomerular basement membrane related**				
*LAMB2*	Laminin β2	AR	Pierson syndrome	(58–60)
*ITGA3*	Integrin α3	AR	Interstitial lung disease, epidermolysis bullosa	(61)
*ITGB4*	Integrin β4	AR	Epidermolysis bullosa	(62)
*COL4A3*	Type IV collagen α3 subunit	AD/AR	Alport syndrome	(63)
*COL4A4*	Type IV collagen α4 subunit	AD/AR	Alport syndrome	(63)
*COL4A5*	Type IV collagen α5 subunit	X-linked	Alport syndrome	(63)

**Nuclear transcription factors and proteins**				
*WT1*	Wilms’ tumor 1	AD	Denys–Drash syndrome, Frasier syndrome	(64, 65)
*LMX1B*	LIM homeobox transcription factor 1β	AD	Nail-patella syndrome	(66, 67)
*SMARCAL1*	SMARCA-like protein	AR	Schimke immune-osseous dysplasia	(68)
*WDR73*	WD repeat domain 73	AR	Galloway–Mowat syndrome	(69)
*LAGE3*	Members of kinase endopeptidase and other proteins of small size (KEOPS) complex	X-linked	Galloway–Mowat syndrome	(70)
*OSGEP*	Members of kinase endopeptidase and other proteins of small size (KEOPS) complex	AR	Galloway–Mowat syndrome	(70)
*TP53RK*	Members of kinase endopeptidase and other proteins of small size (KEOPS) complex	AR	Galloway–Mowat syndrome	(70)
*TPRKB*	Members of kinase endopeptidase and other proteins of small size (KEOPS) complex	AR	Galloway–Mowat syndrome	(70)
*NXF5*	Nuclear RNA export factor 5	X-linked	Cardiac conduction defect	(71)
*NUP93*	Nuclear pore protein 93	AR		(72)
*NUP107*	Nuclear pore protein 107	AR	Galloway–Mowat syndrome like	(73)
*NUP205*	Nuclear pore protein 205	AR		(72)
*XPO5*	Exportin 5	AR		(72)

**Proximal tubule protein reabsorption**				
*CUBN*	Cubilin	AR	Imerslund–Grasbeck syndrome	(74, 75)
*AMN*	Amnionless	AR	Imerslund–Grasbeck syndrome	(74)
*LRP2*	Megalin	AR	Donnai–Barrow/facio-oculo-acoustico-renal syndrome	(76, 77)

**Others**				
*DGKE*	Diacylglycerol kinase ε	AR	Atypical hemolytic uremic syndrome	(78)
*PTPRO*	Glomerular epithelial protein 1	AR		(79)
*PMM2*	Phosphomannomutase 2	AR	Congenital defect of glycosylation	(80)
*ALG1*	B-1,4-mannosyltransferase	AR	Congenital defect of glycosylation	(81)
*EXT1*	Exostosin-1	AD	Multiple exostoses	(5)
*EMP2*	Epithelial membrane protein 2	AR		(7)
*TTC21B*	IFT139	AR		(82)
*NEIL1*	Nei endonuclease VIII-like 1	AR		(83)
*SCARB2*	Lysosomal integral membrane protein type 2	AR	Myoclonus renal failure syndrome	(84)
*ZMPSTE24*	Zinc metalloproteinase STE24	AR	Mandibuloacral dysplasia	(85)
*SGPL1*	Sphingosine-1-phosphate lyase	AR	Ichthyosis, adrenal insufficiency, immunodeficiency, neurological defects	(86)
*FOXP3*	Forkhead box p3	X-linked	Immunodysregulation, polyendocrinopathy, enteropathy, X-linked (IPEX)	(87, 88)

*AR, autosomal recessive; AD, autosomal dominant*.

### Slit Diaphragm Proteins

Nephrin, encoded by *NPHS1*, is an essential component of the slit diaphragm (22). *NPHS1* mutations were originally identified in congenital Finnish type nephrotic syndrome which is characterized by proteinuria that begins *in utero*, premature birth, enlarged placenta, and elevated maternal serum α-fetoprotein levels (23). *NPHS1* mutations are the most common cause of congenital nephrotic syndrome worldwide (21, 24). These patients progress to end-stage renal disease (ESRD) between age 3 and 8 but require unilateral or bilateral nephrectomies and frequent intravenous albumin infusions due to the severity of proteinuria and edema (25). *NPHS1* mutations were also shown to cause SRNS in 7–14% of children and adults (26, 27).

Podocin, encoded by *NPHS2*, is a transmembrane protein that interacts with nephrin and plays an important role in recruitment of nephrin to the slit diaphragm (89). Nephrotic syndrome caused by *NPHS2* mutations has a variable disease course and can cause congenital/infantile nephrotic syndrome or manifest later in childhood or as adult-onset SRNS. NPHS2 mutations are responsible for ~40% of familial SRNS worldwide (28, 29).

Phospholipase C epsilon 1 (PLCε1) is a signaling protein for various G protein-coupled receptors and generates secondary messengers that influence cell growth and differentiation and is thought to be essential for normal glomerular development (30, 90). PLCε1 mutations are the major cause of isolated diffuse mesangial sclerosis occurring in 28–33% of affected families (31).

The other rare forms of slit diaphragm-related proteins associated nephrotic syndrome include CD2-associated protein (CD2AP) (32), an adapter molecule that acts as a bridge between the slit diaphragm and the actin cytoskeleton, and transient receptor potential cation channel type 6 (TRPC6) (33), a non-selective calcium channel in foot processes that interacts with nephrin and podocin. Mutations in TRPC6 are associated with autosomal dominant (AD) SRNS with onset typically in the third or fourth decade of life; however, early childhood onset has also been described (34). FAT atypical cadherin 1 (FAT1) protein colocalizes with nephrin in slit diaphragms and is thought to be a regulator of slit diaphragm–actin cytoskeleton interaction (38, 39). Recently, mutations in *FAT1* were reported to cause SRNS with tubular ectasia, hematuria, and neurological involvement (40).

### Actin Cytoskeleton-Related Proteins

Alpha-actinin 4 (ACTN4) is an actin-binding protein expressed in podocyte foot processes. Mutations in *ACTN4* lead to abnormal adhesion of podocytes to the GBM and are associated with adult-onset SRNS (41, 42). Inverted formin 2 (INF2) regulates actin polymerization and mutations in *INF2* were reported to cause SRNS in adolescence and early adulthood, as well as in patients with Charcot–Marie–Tooth disease (characterized by peripheral neuropathy and FSGS on renal biopsy) (43, 44). Non-muscle myosin 1E (Myo1E) is an actin-binding molecular motor in foot processes and mutations in *MYO1E* cause SRNS in the first decade of life. Electron microscopy (EM) in patients shows characteristic focal thickening, disorganization, and multilamination of the GBM, similar to findings in Alport syndrome (45, 46). Rho GTPases control organization of F-actin in podocytes and their activity is strictly regulated by modifier proteins (91). Mutations in *ARHGAP24* encoding Rho GTPase-activating protein 24 cause familial AD SRNS in the second and third decades of life (48), whereas mutations in *ARHGDIA* encoding RhoGDP dissociation inhibitor α cause congenital nephrotic syndrome or SRNS within the first 2 years of life with associated neurological abnormalities (49). *ANLN* encodes anillin, an actin-binding protein, and was identified as a cause of SRNS due to reduced binding to the slit diaphragm-related protein CD2AP (47). Kidney ankyrin repeat-containing protein (KANK) 1, 2, and 4 were reported to interact with ARHGDIA and regulate RhoGTPase signaling. Mutations in *KANK1, KANK2*, and *KANK4* were reported to cause congenital and early childhood onset nephrotic syndrome (6).

### Mitochondrial Proteins

Coenzyme Q_10_ is a component of mitochondrial inner membrane and plays important roles in electron transport, protection from reactive oxygen species and activating mitochondrial enzymes required in various metabolic pathways (92). Mutations in several genes associated with biosynthesis of coenzyme Q_10_ have been associated with SRNS including *COQ2* (53), *COQ6* (54), *PDSS2* (57), and *ADCK4* (50, 51). The importance of diagnosing mutations in mitochondrial proteins arises from the potential therapeutic benefit with early coenzyme Q_10_ supplementation in these patients (52, 92).

### GBM-Related Proteins

*LAMB2* encodes laminin β2 and its mutations cause Pierson syndrome (congenital nephrotic syndrome-microcoria syndrome) (58). LAMB2 mutations were also reported to cause isolated congenital nephrotic syndrome without ocular abnormalities and nephrotic syndrome within the first decade of life (59, 60). Mutations in *ITGA3* and *ITGB4*, encoding integrin α3 and β4, respectively, were reported to cause congenital nephrotic syndrome associated with epidermolysis bullosa (61, 62). Although genes encoding collagen proteins are known to cause Alport syndrome, mutations in *COL4A3, COL4A4*, and *COL4A5* have also been identified in few familial FSGS patients (childhood and adult onset). However, sensorineural deafness and characteristic electron microscopic abnormalities of the GBM that are seen in Alport syndrome are notably absent in these patients (63).

### Nuclear Transcription Factors and Proteins

Wilms’ tumor 1 (*WT1*) is a tumor suppressor gene that plays an important role in embryonic development of the kidney and genitalia and also regulates nephrin expression in podocytes (13, 64). Mutations in *WT1* were originally described as the cause of Wilms’ tumor, Denys–Drash, and Frasier syndromes. Denys–Drash syndrome is characterized by the presence of Wilms’ tumor, progressive glomerulopathy, and pseudohermaphroditism in patients with 46,XY karyotype (64). Frasier syndrome is characterized by gonadal dysgenesis, gonadoblastoma, and nephrotic syndrome or isolated nephropathy in patients with 46,XY karyotype (64). *WT1* mutations were also shown to be associated with isolated SRNS without tumors or gonadal abnormalities (65). Mutations in *SMARCAL1*, encoding a helicase, cause Schimke immuno-osseous dysplasia which is characterized by growth failure, immune deficiency, and SRNS (68). *LMX1B*, encoding LIM homeobox transcription factor 1β, is required for podocyte differentiation and its mutations typically cause nail-patella syndrome characterized by dystrophic nails, patellar hypoplasia/aplasia with other skeletal abnormalities and SRNS (66), but *LMX1B* mutations were also reported in patients with non-syndromic SRNS (67). Mutations in *WDR73* encoding WD repeat domain 73 was described as the cause for Galloway–Mowat syndrome that is characterized with SRNS and microcephaly with brain anomalies (69). Mutations in genes encoding members of kinase endopeptidase and other proteins of small size (KEOPS) complex (*LAGE3, OSGEP, TP53RK*, and *TPRKB*) were recently described as the cause of Galloway–Mowat syndrome in some families (70). Mutations in *NXF5, NUP93, NUP107, NUP205*, and *XPO5* which are components of nuclear pore complexes and export pathways have been reported to be associated with FSGS (71–73). Recently, mutations in *NUP107* were also reported to cause phenotype similar to Galloway–Mowat syndrome (93).

### Proximal Tubule Protein Absorption Pathway

Cubilin and amnionless, encoded by *CUBN* and AMN, respectively, are subunits of intestinal receptor for vitamin B12/intrinsic factor complex absorption and they are coexpressed with megalin in the proximal tubule of the kidney. Cubilin/amnionless complex and megalin mediate protein reabsorption *via* receptor-mediated endocytosis (94). Albumin and various low-molecular weight proteins are ligands of cubilin/amnionless complex and megalin. *CUBN* and *AMN* mutations were originally described in patients with Imerslund–Gräsbeck syndrome which is characterized by megaloblastic anemia and proteinuria (74). *CUBN* mutations were also described in patients with intermittent nephrotic-range proteinuria without megaloblastic anemia (75). *LRP2* encodes for megalin and its mutations cause Donnai–Barrow/facio-oculo-acoustico-renal syndrome in which proteinuria is among the disease manifestations (76, 77). These discoveries highlight the importance of proximal tubule cubilin/amnionless/megalin reabsorption pathway in reabsorbing proteins that escape the glomerular filtration barrier to produce urine that has little to no albumin or serum proteins.

## Genetic Risk Factors for Idiopathic SSNS and Monogenic Causes of SSNS

The genetic causes and risk loci for SSNS remains elusive, but several *HLA* variants and *PLCG2* variants (a signaling protein that is important for regulation of the immune system) were reported to be associated with increased risk for SSNS in various populations (95). These observations highlight the importance of the immune system in pathogenesis of idiopathic SSNS, although exact genes or risk loci remain to be identified.

Rare monogenic forms of SSNS have been described, although most of these genes were also associated with SRNS in different families. Two children with *PLCE1* mutations were reported to respond to corticosteroid and cyclosporine therapy (30). Epithelial membrane protein 2 (*EMP2*) mutations were identified in patients with childhood onset autosomal recessive SSNS and two Turkish siblings with mutations were reported to have steroid-responsive but frequently relapsing nephrotic syndrome (7). These patients had a sustained remission with cyclophosphamide therapy. However, in the same study, an African-American patient with *EMP2* mutations and MCD on kidney biopsy was reported to be steroid resistant. *EMP2* is thought to regulate caveolin-1 expression which is involved in endocytosis in podocytes. *NPHS1* mutations were identified in patients with SSNS and SRNS who had biopsy-proven MCD (96). A congenital nephrotic syndrome patient with *NPHS1* mutation was also reported to respond partially to steroids and cyclosporine A therapy (24). Mutations in *KANK1* and *KANK2* have been identified in both SSNS and SRNS families (6). Mutations in *EXT1* that encodes exostosin-1 is a cause for AD familial nephropathy and multiple exostoses has been associated with SSNS in an adult patient, but the reported patient also received cyclosporine A and cyclophosphamide in addition to steroids to induce full remission (5). Patients with immune dysregulation, polyendocrinophaty, enteropathy, X-linked (IPEX) syndrome have mutations in the *FOXP3* gene and can develop nephrotic syndrome with MCD or membranous nephropathy (87). There are case reports of patients with IPEX syndrome that demonstrate response to a combination of steroids with cyclosporine A treatment (88).

The hallmark of childhood idiopathic SSNS is a rapid response to steroid therapy which induces complete remission within 6–8 weeks with a single immunosuppressive agent. However, most patients who had a monogenic form of SSNS as described above did not respond to steroids alone and required calcineurin inhibitors to induce remission. This suggests that genetic testing may help to tailor individual therapy in SSNS to start a second immunosuppressive agent sooner than later to minimize steroid exposure. Calcineurin inhibitors were reported to have anti-proteinuric effects due to stabilization of the actin cytoskeleton in podocytes (97), and this may be the main mechanism to induce remission in monogenic SSNS rather than suppression of the immune system.

## Histopathological Findings

Histopathological diagnosis is often obtained when patients do not respond to immunosuppressive therapy, present with renal dysfunction, or have a complicated clinical course. Most common pathological finding in children presenting with nephrotic syndrome is MCD, characterized by little to no changes on light microscopy (LM). Mesangial proliferation, mesangial matrix expansion, increased protein and lipid resorption droplets in tubular epithelial cells, and glomerular hypertrophy are observed on LM. Immunofluorescence (IF) is negative except for occasional IgM and C3 positivity in the mesangium. EM findings include effacement and/or fusion of epithelial foot processes.

Second most common histological finding in children with nephrotic syndrome is FSGS characterized by segmental sclerosis of the glomeruli with glomerular hypertrophy, interstitial fibrosis or tubular atrophy, and endothelial tubuloreticular inclusion bodies on LM. Sclerotic changes occur first in juxtamedullary glomeruli. IF is negative except for occasional IgM and C3 positivity in the mesangium and effacement and/or fusion of foot processes are visible on EM. Morphological characteristics seen on kidney biopsy cannot usually distinguish genetic and non-genetic forms of FSGS with some exceptions such as Alport’s syndrome where splitting of the GBM is a unique pathognomonic finding (2). There are five morphological variants of the lesions of FSGS based on LM but EM findings are similar in all subtypes (98). The histological variants of FSGS include FSGS not otherwise specified, collapsing variant, tip variant, perihilar variant, and cellular variant. The impact of histological variants of FSGS on renal prognosis and response to therapy is yet to be fully investigated. Other less common histopathological findings in patients with nephrotic syndrome include membranoproliferative and mesangioproliferative glomerulonephritis, membranous nephropathy, focal and global glomerulosclerosis, and IgA and IgM nephropathy.

## Treatment and Prognosis

Most children who present with nephrotic syndrome respond to high dose steroid therapy, but ~20% of children are diagnosed with SRNS after a trial of steroid therapy for 6–8 weeks. Children may achieve partial or complete remission within a few days of initiating therapy but steroid treatment is continued for several weeks to prevent frequent relapses (2). Approximately 50–70% of patients will have relapsing and remitting disease that is usually responsive to reinstating steroid therapy. Maintenance immunosuppression with alternate immunosuppressive agents is required in children with frequent relapses to minimize steroid exposure and its adverse effects. Children diagnosed with SRNS often respond to angiotensin-converting enzyme (ACE) inhibitor therapy (99), calcineurin inhibitors (100–103), or other immunosuppressive agents (104). In a recent analysis of the PodoNet registry cohort, more than two-third of patients with SRNS responded to immunosuppressive therapy including those with known genetic mutations. Confirmation of a genetic diagnosis but not the histopathological disease type was strongly predictive of response to immunosuppressive therapy (8). However, treatment of monogenic nephrotic syndrome with immunosuppression has not been carefully examined by large clinical studies and little is known about genotype-to-phenotype correlation for most genetic mutations. With current state of evidence, histopathological diagnosis and genetic testing are not required to initiate treatment of either SSNS or SRNS and no specific recommendations for treatment can be made based on genetic diagnosis (3). Prognosis is excellent for SSNS with <3% developing ESRD as opposed to patients with SRNS who have increased risk of ESRD needing dialysis and/or kidney transplantation (8).

## Challenges to Genetic Testing

In clinical practice, gene sequencing is not routinely performed for all patients who carry the diagnosis of nephrotic syndrome. Factors that are taken into consideration by the ordering physician include cost, access, and availability of gene sequencing, patient/family interest, and whether test results will aid in diagnosis, management, and determining prognosis for the patient. Currently, there are no clear guidelines for performing genetic tests in patients with nephrotic syndrome. Clinicians face several challenges to performing mutation analysis including lack of genotype-to-phenotype correlations for several of the known gene mutations and in many instances, clinical management is unlikely to change given positive genetic test results. Second, a negative result does not exclude monogenic cause of nephrotic syndrome as many of the gene test panels do not include the complete set of identified genes and many more novel genes are being identified annually. Whole exome sequencing is not readily available to most clinicians and has its own drawbacks (105). Despite these challenges, genetic testing is particularly useful in certain clinical situations. Early onset of disease and family history of nephrotic syndrome were the most important risk factors for finding pathogenic mutations in large clinical studies of adult and pediatric patients with nephrotic syndrome (8, 11, 106). A positive test result for certain gene mutations has been shown to be associated with SRNS and knowledge of this information will help clinicians to avoid a trial of steroid therapy in such patients, determine renal prognosis and for genetic counseling of families (65, 107). Anecdotal reports have also shown certain genetic mutations causing nephrotic syndrome are responsive to non-steroidal agents such as cyclosporine, ACE inhibitors, and coenzyme Q_10_ supplementation (30, 52, 87, 88, 92, 108, 109). However, large studies are lacking to comprehensively evaluate all gene mutations identified in monogenic nephrotic syndrome to accurately predict response to treatment. Gene testing has also been useful for pre-transplant evaluation of patients with nephrotic syndrome to predict risk of recurrence and to guide post-transplant management. Specific examples include patients with *NPHS2* mutations do not respond to steroid therapy and are less likely to have post-transplant recurrence of FSGS (110) but such predictions are not available for all gene mutations. Gene sequencing poses significant challenges in evaluating potential living related donors for patients with familial nephrotic syndrome and its utility is controversial. AD, AR and X-linked gene mutations confer different risk profiles for donors and are influenced by factors such as gene penetrance, modifier genes, epigenetic factor, and environmental factor. Therefore, large scale prospective clinical studies are urgently needed and must include genetic testing for better patient stratification to establish diagnosis, determine choice of immunosuppression, predict post-transplant diseases course and treatment, selection of living donors in familial cases, and to determine prognosis.

## Conclusion

Nephrotic syndrome in children is easy to diagnose but challenging to treat due to its complex etiology and mechanisms by which the glomerular filtration barrier is disrupted to induce proteinuria. A renal biopsy is not indicated to diagnose or initiate treatment as recent studies demonstrate that a majority of patients respond to immunosuppressive therapy regardless of histopathological diagnosis. A traditional approach was to perform genetic testing in those patients who are likely to not respond to immunosuppression (e.g., familial cases and congenital nephrotic syndrome) to spare them from futile therapies that can cause serious adverse effects. However, as genetic testing becomes more prevalent it is increasingly evident that a subset of patients with monogenic causes of SSNS and SRNS will respond to immunosuppression and other anti-proteinuric therapies to achieve partial or complete remission. As genetic testing becomes more prevalent and affordable, we expect rapid advances in our understanding of mechanisms of proteinuria creating an opportunity to personalize treatment in the future with a “precision medicine” approach for both adults and children with nephrotic syndrome.

## Author Contributions

OC and FP contributed equally to the writing of this manuscript.

## Conflict of Interest Statement

The authors declare that the research was conducted in the absence of any commercial or financial relationships that could be construed as a potential conflict of interest.
